# Different conformational dynamics of SNARE protein Ykt6 among yeast and mammals

**DOI:** 10.1016/j.jbc.2023.104968

**Published:** 2023-06-26

**Authors:** Jie Ji, Yiping Yu, Shaowen Wu, Dongdong Wang, Jingwei Weng, Wenning Wang

**Affiliations:** 1 Department of Chemistry, Institute of Biomedical Sciences and Multiscale Research Institute of Complex Systems, Fudan University, Shanghai, China; 2 Guangdong Key Laboratory for Crop Germplasm Resources Preservation and Utilization, Agro-biological Gene Research Center, Guangdong Academy of Agricultural Sciences, Guangzhou, Guangdong, China; 3 DP Technology, Beijing, China

**Keywords:** SNARE, Ykt6, protein conformation, smFRET, MD simulation

## Abstract

Ykt6 is one of the most conserved SNARE (N-ethylmaleimide-sensitive factor attachment protein receptor) proteins involved in multiple intracellular membrane trafficking processes. The membrane-anchoring function of Ykt6 has been elucidated to result from its conformational transition from a closed state to an open state. Two ways of regulating the conformational transition were proposed: the C-terminal lipidation and the phosphorylation at the SNARE core. Despite many aspects of common properties, Ykt6 displays differential cellular localizations and functional behaviors in different species, such as yeast, mammals, and worms. The structure–function relationship underlying these differences remains elusive. Here, we combined biochemical characterization, single-molecule FRET measurement, and molecular dynamics simulation to compare the conformational dynamics of yeast and rat Ykt6. Compared to rat Ykt6 (*r*Ykt6), yeast Ykt6 (*y*Ykt6) has more open conformations and could not bind dodecylphosphocholine that inhibits *r*Ykt6 in the closed state. A point mutation T46L/Q57A was shown to be able to convert *y*Ykt6 to a more closed and dodecylphosphocholine-bound state, where Leu46 contributes key hydrophobic interactions for the closed state. We also demonstrated that the phospho-mutation S174D could shift the conformation of *r*Ykt6 to a more open state, but the corresponding mutation S176D in *y*Ykt6 leads to a slightly more closed conformation. These observations shed light on the regulatory mechanism underlying the variations of Ykt6 functions across species.

The N-ethylmaleimide-sensitive factor attachment protein receptor (SNARE) proteins are involved in the fusion of vesicles with their target membranes (1, 2). It plays an important role in the normal growth of cells and intracellular signal transduction such as the synthesis and transport of biological molecules within the cell (3). During the docking process of the vesicle and the target membrane, one SNARE protein located on the vesicle membrane will assemble with three homologous complementary SNARE proteins on the target membrane to form a zipper-like SNARE complex, which mediates the fusion of the membrane (4). The central layer (the "0" layer) of the four-helix bundle of the SNARE complex consists of three polar amino acids (glutamine Q) and one positively charged amino acid (arginine R), so SNAREs are classified as R-SNAREs and Q-SNAREs (5). Generally, R-SNAREs act in conjunction with the vesicle, and therefore are also referred to as v-SNAREs, which are incorporated into the membranes of transport vesicles during the initiation of membrane fusion. Q-SNAREs are generally associated with the target membrane, so they are also called t-SNAREs (6).

Ykt6 is one of the most conserved R-SNAREs in eukaryotes (7). It is composed of three domains: an N-terminal longin domain, a conserved central 60 to 70 amino acid ‘‘SNARE core’’ that mediates the self-assembly of the four-helix-bundle SNARE core complex, and a C-terminal “CCXIM” motif (8). It is generally believed that mammalian Ykt6 protein function is regulated by two lipid modifications, that is, the palmitoylation/geranylgeranylation at the first cysteine and the farnesylation at the second cysteine of the “CCAIM” motif (9). Farnesylation of Cys195 in mammalian Ykt6 is irreversible, while the palmitoylation is reversible and responsible for the conformational transition from the closed (inactive) to the open (active) state (10, 11). Previous studies claimed that the soluble inactive self-inhibited conformation of mammalian Ykt6 is stabilized by the farnesyl group sandwiched between the SNARE and the longin domains (12). Dodecylphosphocholine (DPC) is found to be a good surrogate for the farnesyl group of *r*Ykt6 *in vitro* (13). After binding with DPC, the core of *r*Ykt6 SNARE forms four helices wrapping around the longin domain, and the whole protein is in a closed conformation (13, 14). The structure of *r*Ykt6/DPC complex (Protein Data Bank-ID: 3KYQ) is considered to mimic the farnesylated closed form of Ykt6 (13). Besides the lipidation, it was recently found that the conformation of Ykt6 could also be regulated by phosphorylation (15, 16, 17). Phosphorylation at the evolutionarily conserved site S174 in human Ykt6 (*h*Ykt6) drives the conversion from a closed cytosolic to an open membrane-bound state (17). In yeast, Ykt6 is directly phosphorylated by Atg1 kinase at the T158, S182, and S183 sites, leaving *y*Ykt6 in an inactive state preventing the formation of the SNARE complex and premature autophagosome-vesicle fusion (16).

Although Ykt6 is highly conserved in sequence (Fig. 1*A*), functional differences have been reported among different species. In yeast, a majority of *y*Ykt6 are membrane-localized through the lipid groups on the “CCIIM” motif (18, 19), while the mammalian Ykt6 is almost uniformly distributed throughout the cytoplasm and often extends into larger neurites (17, 20). Recent study of the regulatory role of phosphorylation on Ykt6 showed that the phospho-mutations yielded different effects in yeast *versus* dopaminergic neurons in *Caenorhabditis elegans* (17). To explore the molecular mechanism of the functional differences of Ykt6 across yeast to mammals, we investigated the conformational dynamics of *y*Ykt6 and compared it with *r*Ykt6.

**Figure 1:**
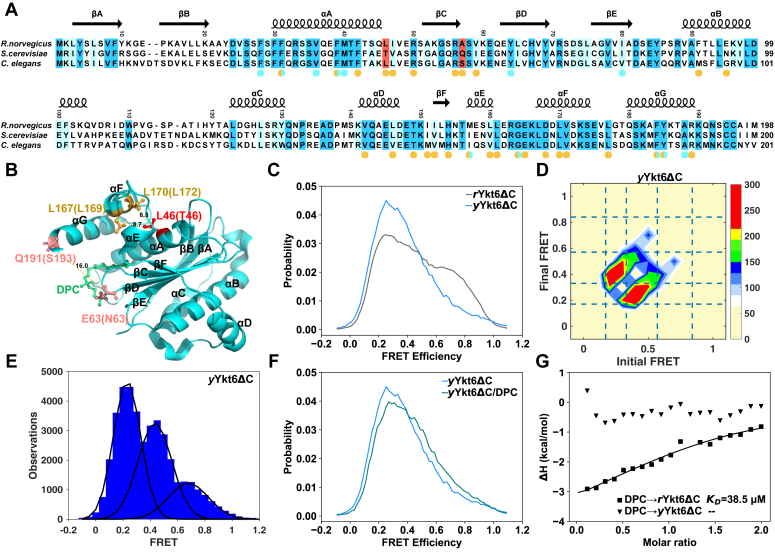
***y*Ykt6ΔC adopts more open conformation than *r*Ykt6ΔC.***A*, sequence alignment of *y*Ykt6, *r*Ykt6, and *Caenorhabditis elegans* Ykt6 with secondary structure elements based on the crystal structure of *r*Ykt6 (3KYQ). Residues 46 and 57 (48 and 59 in *C. elegans*) are highlighted in *red*. The completely conserved residues are highlighted in *blue*, and the highly conserved residues in *light blue*. Residues involved in the longin/SNARE core interactions are indicated with *orange circles*. Residues involved in DPC/farnesyl group binding are indicated with *cyan circles*. *B*, structure diagram of *r*Ykt6 showing the positions of labeled fluorescence dyes (*dark pink*). DPC is highlighted in *green*. The key hydrophobic amino acids are highlighted in *dark yellow* or *red*. The numbers in parentheses denote the corresponding residues in *y*Ykt6. The *yellow dashed line* represents the inter-residue distances (*d*_63-191_, *d*_46-167_, and *d*_46-170_). *C*, FRET efficiency distribution profiles of *r*Ykt6ΔC (*gray*) and *y*Ykt6ΔC (*blue*). *D*, TDP compiles all the transitions from the initial to the final FRET efficiency of *y*Ykt6ΔC system indicating three major conformational states. *E*, FRET efficiency histogram of *y*Ykt6ΔC based on HMM analysis. *F*, FRET efficiency distribution profiles of Ykt6ΔC (*blue*), *y*Ykt6ΔC/DPC (*teal*). *G*, ITC measurements of the interaction between DPC and *y*Ykt6ΔC/*r*Ykt6ΔC. DPC, dodecylphosphocholine; HMM, hidden Markov model; ITC, isothermal titration calorimetry; TDP, transition density map.

We previously reported the conformational dynamics of *r*Ykt6 in detail, revealing that DPC binding shifts the structure to a more closed state (14). In this work, we examined the conformational dynamics of *y*Ykt6 through single-molecule Förster resonance energy transfer (smFRET) measurements and compared the conformational difference between *y*Ykt6 and *r*Ykt6, finding that *y*Ykt6 adopts more open conformations than *r*Ykt6 does and could not bind to DPC. The conformation equilibrium of *y*Ykt6 could be shifted to the closed state by point mutation T46L/Q57A, which also enables DPC binding. The effects of phospho-mutations at the conserved sites S174/S176 of *r*Ykt6/*y*Ykt6 on their conformations were also explored.

## Results

### Yeast Ykt6 adopts a more open conformation than rat Ykt6

We employed smFRET to explore the conformational dynamics of *y*Ykt6ΔC (*y*Ykt6 without the “CCIIM” motif, residues 1–195). A pair of cysteine residues was introduced at Asn63 and Ser193 by mutations, while the native Cys76 was mutated to serine (Fig. 1*B*). Purified *y*Ykt6ΔC-N63C/C76S/S193C showed a similar elution profile on size-exclusion chromatography (SEC) to that of the WT *y*Ykt6ΔC (Fig. S1). Then, we performed smFRET measurements of dye-labeled *y*Ykt6ΔC on a laboratory-built setup (see Experimental procedures for more details). The typical lifetime of the dye attached to *y*Ykt6ΔC is about 20 s and the longest trajectory is about 50 s (Fig. S2*A*). The FRET efficiency distribution is broad and could not be fitted with one single Gaussian (Fig. 1*C*). In comparison with *r*Ykt6ΔC (14), *y*Ykt6ΔC exhibits a relatively open conformational distribution with a high proportion of low FRET efficiency (Fig. 1*C*). The trajectories exhibited multiple distinct levels of FRET efficiency, suggesting conformational transitions in *y*Ykt6ΔC (Fig. S2*A*). To determine the number of conformational states, we performed a hidden Markov model (HMM) analysis of the smFRET trajectories using the vbFRET method (21). The optimal number of states was determined to be three (Fig. S2*B*). The three conformational states from open to closed state were named as E1, E2, and E3. The transition density map (the initial and final HMM FRET efficiency of each transition) shows that the transition probability between E1 and E2 is remarkably higher than that of the E2 ↔ E3 transition, while direct transition between E1 and E3 was hardly observed (Fig. 1*D*). The threshold algorithm based on HMM analysis revealed that the most probable FRET efficiencies and the state populations of E1, E2, and E3 of *y*Ykt6ΔC are 0.20, 0.42, 0.68 and 50.7%, 39.7%, 10.2%, respectively (Table S1). The E1 state with FRET efficiency of 0.20 accounts for the majority of the population, and there is no state with the most probable FRET efficiency higher than 0.7 (Fig. 1*E*). In comparison, *r*Ykt6ΔC adopts five conformational states (E1, E2, E3, E4, and E5) with FRET efficiencies of 0.20, 0.35, 0.48, 0.64, 0.79 and populations of 31.4%, 19.8%, 19.3%, 16.2%, and 13.3%, respectively (Table S1) (14). Obviously, the conformation of *y*Ykt6ΔC was more open than *r*Ykt6ΔC.

Although *y*Ykt6ΔC generally adopts more open conformations than *r*Ykt6ΔC does, the C-terminal lipidation may regulate the conformational distribution. To examine the effect of lipid groups, we employed DPC as farnesyl group mimics (13). Comparison of the FRET efficiency distributions of *y*Ykt6ΔC in the presence and absence of DPC demonstrates that the distribution is only slightly affected by addition of 2 mM DPC (Fig. 1*F*). This is in contrast to the case of *r*Ykt6ΔC, where DPC significantly shifted the FRET efficiency distribution toward the higher values (14). We then examined the interaction between *y*Ykt6ΔC and DPC by isothermal titration calorimetry (ITC) measurements. It turns out that DPC could not bind to *y*Ykt6ΔC (Fig. 1*G*). In comparison, DPC binds *r*Ykt6ΔC with a *K*_D_ of 38.5 μM (Fig. 1*G*). Therefore, *y*Ykt6ΔC has a more open conformation than *r*Ykt6ΔC, and the C-terminal lipid group might not be able to regulate its conformation effectively.

### Mutations at the longin-SNARE interface lead to more closed conformation of *y*Ykt6ΔC

**Figure 2:**
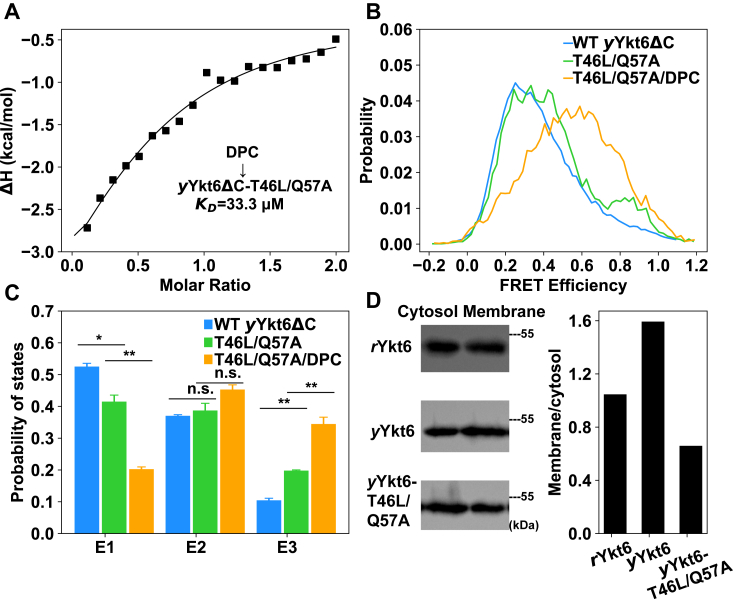
**Point mutations lead to more closed conformation and DPC binding ability of *y*Ykt6.***A*, ITC measurements of the interaction between DPC and *y*Ykt6ΔC-T46L/Q57A mutant. *B*, FRET efficiency distribution profiles of WT *y*Ykt6ΔC (*blue*), T46L/Q57A mutant (*green*), and T46L/Q57A/DPC (*orange*) systems. *C*, comparison of the populations of conformational states of WT *y*Ykt6ΔC (*blue*), T46L/Q57A mutant (*green*), and T46L/Q57A/DPC systems (*orange*). Significance was determined by the two-tailed *t* test. ∗*p* < 0.05, ∗∗*p* < 0.01. *D*, fractionations of subcellular localizations of WT *r*Ykt6, WT *y*Ykt6, and *y*Ykt6-T46L/Q57A mutant. The membrane/cytosolic ratios of WT Ykt6 and mutant were quantified. DPC, dodecylphosphocholine; ITC, isothermal titration calorimetry.

According to the crystal structure of *r*Ykt6ΔC/DPC complex (13), the DPC-binding pocket is located at the interface of the longin domain and the SNARE core (Fig. 1*B*). Therefore, we speculated that some key residues at this interface may determine the conformational distribution of Ykt6, as well as the DPC binding affinity. We generated several mutations at the interface and found that a double mutation T46L/Q57A of *y*Ykt6ΔC could greatly enhance the binding of DPC to *y*Ykt6ΔC with a *K*_D_ of 33.3 μM, similar to that between DPC and *r*Ykt6ΔC (Figs. 2*A* and 1*G*).

The smFRET measurements showed that the FRET efficiency distribution of *y*Ykt6ΔC-T46L/Q57A mutant was shifted to higher values with respect to WT *y*Ykt6ΔC, suggesting that the mutant adopts more closed conformations (Fig. 2*B*). The HMM analysis revealed that the population of the most closed state E3 in T46L/Q57A mutant increased and the population of the E1 state decreased (Fig. 2*C*). Moreover, the T46L/Q57A mutant showed a larger elution volume than WT on SEC, suggesting a more compact conformation of the mutant (Fig. S3). Then, we examined the effect of DPC binding on the conformational distribution of the T46L/Q57A mutant. As expected, addition of DPC shifted the FRET efficiency distribution of *y*Ykt6ΔC-T46L/Q57A to higher values (Figs. 2, *B* and *C* and S4, Table S2), indicating that DPC could further shift the conformation of *y*Ykt6ΔC-T46L/Q57A to more closed states. Altogether, these results demonstrated that enhancing the interaction between the longin domain and the SNARE core of *y*Ykt6 could shift its conformation to a more closed state and empower it to bind DPC.

### Different cellular localizations of *y*Ykt6, *y*Ykt6-T46L/Q57A, and *r*Ykt6

In order to examine the relevance of protein conformations and cellular localizations, we expressed GFP-tagged full-length *y*Ykt6, *y*Ykt6-T46L/Q57A, and *r*Ykt6 in 293T cells or HeLa cells. It turns out that WT *y*Ykt6 is mainly localized at the perinuclear regions, which have been suggested to be the Golgi apparatus and punctuate organelle membranes (19) (Fig. S5). This localization is consistent with the above *in vitro* experiments, which indicated a relatively open conformation of *y*Ykt6 and the inability of lipids to restrain the conformation in a closed state. On the other hand, both *r*Ykt6 and *y*Ykt6-T46L/Q57A mutant showed a diffused pattern of localization throughout the cytosol and nucleus (Figs. 2D and S5), indicating a more compact and closed autoinhibitory conformation. Overall, these results suggest that the distinct conformational distributions of *y*Ykt6, *y*Ykt6-T46L/Q57A, and *r*Ykt6 revealed by the *in vitro* experiments are directly related to their cellular localizations, suggesting that there might be functional differences of Ykt6 in rats and yeast.

### Molecular dynamics simulations revealed details that determine the conformational states

**Figure 3:**
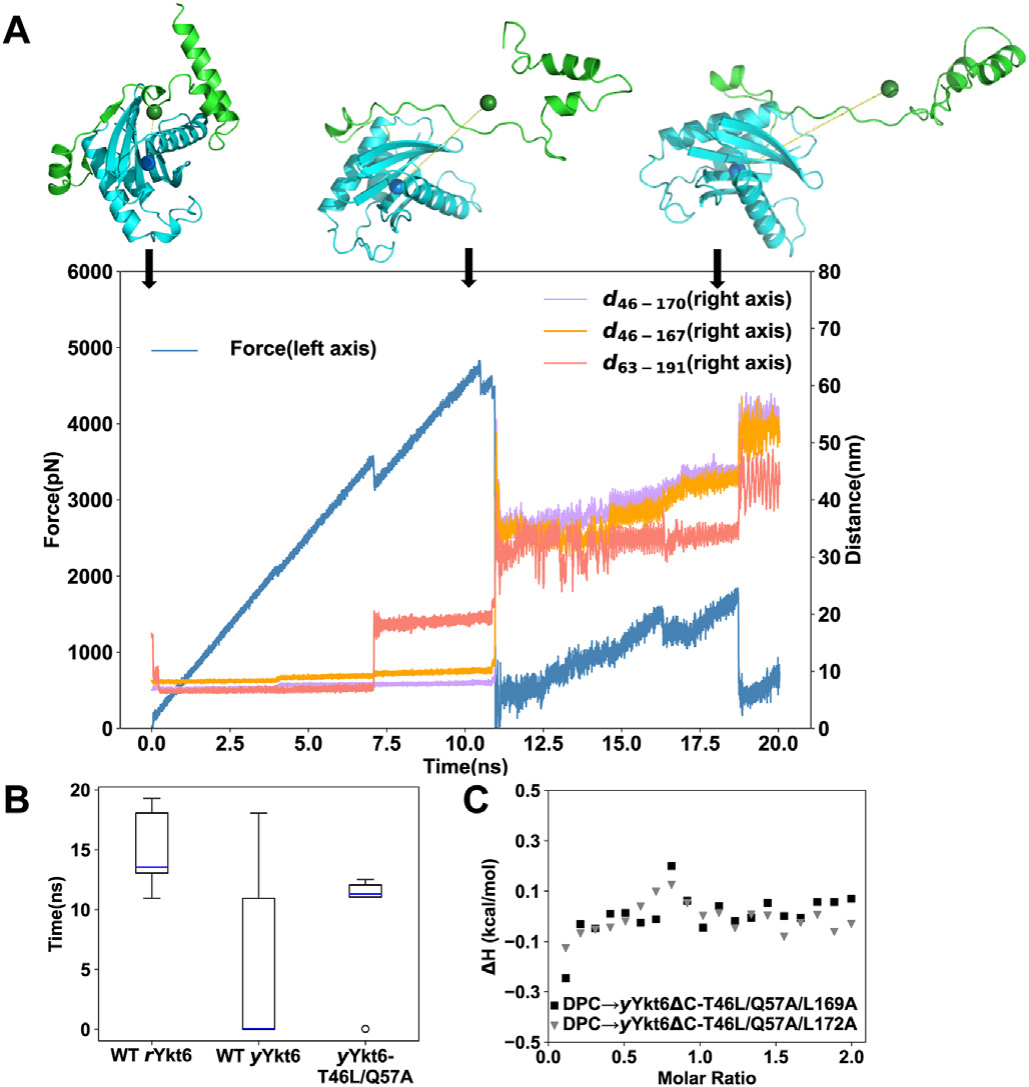
**The hydrophobic contacts between αF and L46 are crucial for the closed conformation and DPC binding.***A*, time variations of SMD force and the inter-residue distances *d*_46-170_, *d*_46-167_, and *d*_63-191_ in WT *r*Ykt6 system. Three structures representing the beginning point, turning point, and end point along the SMD trajectory are shown (*green* for the SNARE core, *blue* for the longin domain). The fixed (*blue*) and pulling (*green*) centers are shown as *spheres*, and connected with a *yellow dashed line*. *B*, statistics of the times of the critical turning points in WT *r*Ykt6, WT *y*Ykt6, and *y*Ykt6-T46L/Q57A mutant systems. *C*, ITC measurements of binding between DPC and *y*Ykt6ΔC-T46L/Q57A/L169A or *y*Ykt6ΔC-T46L/Q57A/L172A. DPC, dodecylphosphocholine; ITC, isothermal titration calorimetry; SMD, steered molecular dynamics.

In order to explore the molecular details that determine the conformational states of Ykt6, we performed constant-velocity steered molecular dynamics (SMD) simulations to monitor the closed-to-open conformational transitions of *r*Ykt6, *y*Ykt6, and *y*Ykt6-T46L/Q57A systems (see Experimental procedures for details). The whole SNARE core was pulled away with the constant-velocity mode SMD implemented in the simulation package NAMD (https://www.ks.uiuc.edu/Research/namd/), in which we fixed atoms of the longin domain (*r*Ykt6 residue 1–131, *y*Ykt6 residue 1–133), and pulled atoms of SNARE (*r*Ykt6 residue 132–198, *y*Ykt6 residue 134–200) along the vector from the centroid of the longin domain toward the centroid of the SNARE core at a constant velocity (22). In all the three systems, the steer forces increased almost linearly from the beginning of the simulations and dropped abruptly to near zero between 10 and 15 ns (Figs. 3*A* and S6). Around this critical turning point of steer force, the SNARE core domain dissociated from the longin domain and the initially compact conformation of Ykt6 turned into a relatively open state (Fig. 3*A*). On average, the turning points of the steer forces in *r*Ykt6 and *y*Ykt6-T46L/Q57A systems appeared later than that in *y*Ykt6 (Fig. 3*B*), suggesting that the interactions between the longin and the SNARE core are stronger in *r*Ykt6 and *y*Ykt6-T46L/Q57A systems. This observation is consistent with the above experimental results.

According to the crystal structure of *r*Ykt6/DPC complex (13), the compact conformation of *r*Ykt6 is maintained by the interactions among fragments including αA (residue 30–50), αF (residue 169–175), αG (residue 181–192), βC (residue 54–60), and βF (residue 153–158) (Fig. 1*B*). In order to identify the key interactions that determine the compact conformation, we examined various inter-residue distances among these fragments along the SMD trajectories, and identified two inter-residue distances, *d*_46-170_ (*d*_46-172_ in *y*Ykt6) and *d*_46-167_ (*d*_46-169_ in *y*Ykt6) that exhibit synchronous abrupt changes at the critical turning point of the steer force (Figs. 3*A* and 1*B*). This is in line with the mutagenesis results showing that L46 is of crucial importance.

We also examined the distance between the two fluorophore-labeled residues, that is, *d*_63-191_ (*d*_63-193_ in *y*Ykt6) and found that it generally synchronized with the variations of the steer force (Figs. 3*A* and 1*B*). Before the critical turning point, the steer force exhibited a small drop around 7 ns in most of the trajectories (Figs. 3*A* and S6). This corresponds to the first surge of *d*_63-191_ (*d*_63-193_ in *y*Ykt6), indicating the dissociation of the αG helix from the longin domain (Fig. 3*A*). In the second stage, the steer force dropped to zero and *d*_46-170_ and *d*_46-167_ (*d*_46-172_, *d*_46-169_ in *y*Ykt6) surged abruptly, indicating the dissociation of the αF helix from the longin domain (Fig. 3*A*). This feature of the steer force profile suggests that the dissociation of the αF helix is the rate-limiting step of the conformation opening process. The hydrophobic residues, such as L167 and L170 on αF, might also be important for the binding between the SNARE core and the longin domain. In the third stage, SMD force still accumulated gradually, which could be attributed to the stable interactions between βC and βF strands.

To verify the role of L167/L170 (L169/L172 in yeast) observed in the SMD simulations, we mutated these two residues to Ala or Glu in *r*Ykt6 and *y*Ykt6ΔC-T46L/Q57A mutant. ITC measurements showed that these mutations disrupt the binding with DPC (Figs. 3*C* and S7), indicating the importance of these hydrophobic residues on αF.

### Comparison of the role of phosphorylation in the conformational dynamics of *r*Ykt6ΔC and *y*Ykt6ΔC

**Figure 4:**
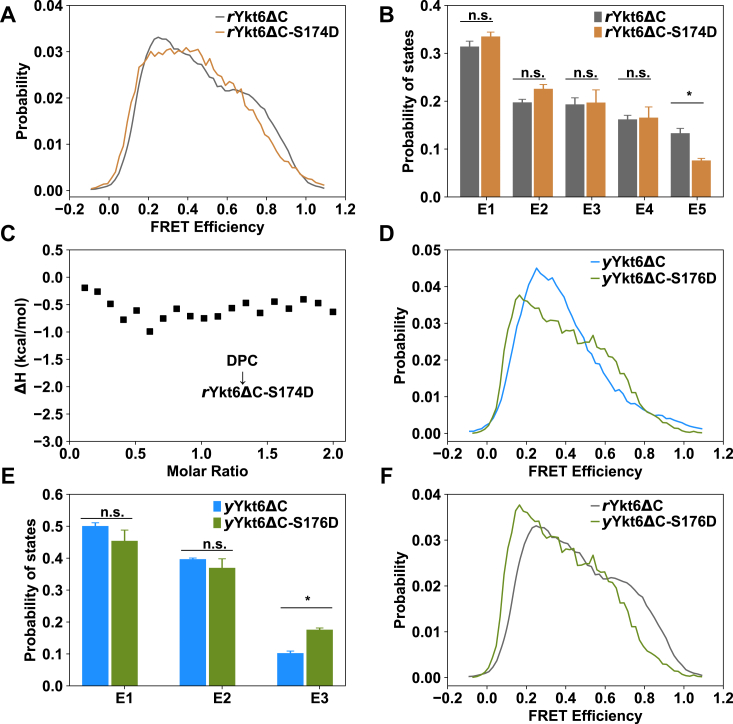
**The effect of phospho-mutations on the conformations of Ykt6ΔC.***A*, FRET efficiency distribution profiles of *r*Ykt6ΔC (*gray*) and *r*Ykt6ΔC-S174D (*brown*) systems. *B*, comparison of the distribution ratios of conformational states of *r*Ykt6ΔC (*gray*) and *r*Ykt6ΔC-S174D (*brown*). *C*, ITC measurement of the interaction between DPC and *r*Ykt6ΔC-S174D. *D*, FRET efficiency distribution profiles of *y*Ykt6ΔC (*blue*) and *y*Ykt6ΔC-S176D (*olive drab*). *E*, comparison of the population ratios of the conformational states of *y*Ykt6ΔC (*blue*) and *y*Ykt6ΔC-S176D (*olive drab*). *F*, FRET efficiency distribution profiles of WT *r*Ykt6ΔC (*dim gray*) and *y*Ykt6ΔC-S176D (*olive drab*). Significance was determined by the two-tailed *t* test. ∗*p* < 0.05, ∗∗*p* < 0.01. DPC, dodecylphosphocholine; ITC, isothermal titration calorimetry.

It was recently reported that an evolutionarily conserved residue in the SNARE core of Ykt6 (S174 in *r*Ykt6 and S176 in *y*Ykt6) could be phosphorylated, and the phosphorylation triggers the conformation to a more open state (17). As we have found the different conformational distributions between *r*Ykt6ΔC and *y*Ykt6ΔC, the next question is how phosphorylation of S174/S176 affects the conformational states of *r*Ykt6ΔC and *y*Ykt6ΔC, respectively. We first performed smFRET measurements to examine the conformational changes upon phospho-mutations. Comparison of the measurements of WT *r*Ykt6ΔC and *r*Ykt6ΔC-S174D showed that the S174D mutation slightly shifts the FRET efficiency distribution toward the lower values (Fig. 4, *A* and *B*). The population of the E5 state (the most closed one) decreased with respect to WT *r*Ykt6ΔC (Fig. 4*B*). Moreover, addition of DPC hardly disturbed the FRET efficiency distribution of *r*Ykt6ΔC-S174D (Fig. S8). In line with this, ITC experiments showed that DPC could not bind to *r*Ykt6ΔC-S174D mutant, while it binds to WT *r*Ykt6ΔC with a *K*_D_ of 38.5 μM (Figs. 4*C* and 1*F*). Therefore, the phospho-mutation S174D slightly shifts the conformations of *r*Ykt6ΔC to a more open state and disrupts the binding of DPC.

We also compared the smFRET measurements of WT *y*Ykt6ΔC and *y*Ykt6ΔC-S176D. It is surprising that S176D slightly shifts the FRET distribution to higher values and the proportion of the closed conformation E3 increased (Fig. 4, *D* and *E*). As mentioned above, WT *y*Ykt6ΔC could not bind DPC, and here ITC measurements showed that the phospho-mutant *y*Ykt6ΔC-S176D could not bind DPC either (Fig. S9). By comparing the smFRET results of *y*Ykt6ΔC-S176D and *r*Ykt6ΔC, we could find that the conformation of *y*Ykt6ΔC-S176D is still much more open than that of *r*Ykt6ΔC (Fig. 4*F*). This is in line with the ITC experiment that DPC could not bind *y*Ykt6ΔC-S176D either (Fig. S9). WT *y*Ykt6ΔC itself is in a relatively open conformation. Although phosphorylation at S176 shifts it toward a slightly more closed one, the overall conformation is more open than *r*Ykt6ΔC, and DPC has no interaction with the protein.

## Discussion

The conformational state of the longin SNARE Ykt6 is tightly related to its cellular localization and function. It has been revealed that the conformational state could be regulated by the lipid modification (11, 22) and phosphorylation (15, 16, 17). However, it is intriguing that the function and conformational regulation of Ykt6 exhibit differences among various species (17, 18, 19, 20, 23). Although it is widely recognized that the C-terminal lipidation at the second cysteine residue of the “CCXIM” motif results in a self-inhibition conformation, our experiments demonstrated that yeast Ykt6 might be an exception. *y*Ykt6ΔC adopts a more open conformation than *r*Ykt6ΔC and could not bind DPC. Accordingly, *y*Ykt6 showed major membrane localization in cells (Fig. 2). The more open conformation of *y*Ykt6 with respect to *r*Ykt6 could be traced to their sequence difference. Despite the high sequence conservation between *y*Ykt6 and *r*Ykt6, a few key residues at the hydrophobic core of the longin-SNARE interface could make a difference. As we have shown, the T46L/Q57A double mutation converted the open conformation of WT *y*Ykt6 to a more closed one and empowers it to bind DPC. Thr/Leu46 on αA helix of Ykt6 is not a conserved site, but the hydrophobic nature of residue in *r*Ykt6 is crucial for the binding of αF to the longin domain through interaction with Leu170 and Leu167, thereby maintaining the closed conformation. Although Leu46 does not make contact with DPC directly in the closed structure, it apparently facilitates lipid binding. This suggests that the packing of the αF helix with the longin domain is important for forming the lipid-binding pocket. Therefore, it is most likely that lipid modification in *y*Ykt6 lacks the ability of regulating the conformational state.

The phosphorylation at the evolutionarily conserved site Ser174 was previously shown to trigger the conformational opening of human Ykt6 (17). Here, the same role of phosphorylation at Ser174 in *r*Ykt6 was also validated through the smFRET experiments, and the conformation of phospho-mutant S174D could not be regulated by DPC (Fig. S8). However, the phosphorylation of the conserved site Ser176 in *y*Ykt6 does not play the same role as that in mammalian Ykt6. The S176D mutant exhibits slightly more closed conformation than the WT *y*Ykt6, but DPC could not bind to it either, most likely due to the intrinsically more open conformation of S176D than that of *r*Ykt6. Taken these lines of evidence together, we could speculate that phosphorylation triggers the conformational opening and facilitates membrane insertion of mammalian Ykt6 in addition to providing a negatively charged group at Ser174, but phosphorylation at the same site of *y*Ykt6 does not affect its membrane localization. The main role of phosphorylation in *y*Ykt6 is introducing a phosphor group, which affects the partner binding of *y*Ykt6. Given this perspective, the different functional consequences of phosphorylation of Ser176/Ser177 in yeast and worm (17) may result from the inherent differences of *y*Ykt6 and worm Ykt6. In the sequence of the worm Ykt6, the position 48 is a Leu as that in mammalian Ykt6 (Fig. 1*A*), thereby its conformation is most likely as closed as *r*Ykt6. Consequently, the open conformation dephosphorylation mutant S177A behaves differently from the WT in *C elegans*, while in yeast the S177A mutant behaves more like the WT (17). In other words, phosphorylation of Ser174/Ser177 regulates both conformational state of Ykt6 and its protein binding property in mammals and *C. elegans*, but phosphorylation only affects the protein binding capacity of Ykt6 in yeast. Besides Ser174, several other phosphorylation sites on Ykt6 have been identified (15, 16), we anticipate that more regulatory mechanisms and delicate differences across species are yet to be discovered.

Although Ykt6 is a highly conserved SNARE from plants to animals, the functional regulation mechanisms are finely tuned by small sequence variations. Inspired by the studies of WASP family proteins (24, 25), we conducted sequence alignments of Ykt6 orthologs spanning various eukaryotic organisms and subsequently constructed a phylogenetic tree (Fig. S10). Notably, the Ykt6 orthologs manifest three major branches (Fig. S10*A*). The branch containing Ykt6 from protist organisms distinctly separates them from their counterparts in more advanced organisms. Similarly, the branch encompassing Eumycetes and Plantae organisms clearly distinguishes them from Animalia (Fig. S10*A*). Interestingly, residue 46 (or its equivalents) remains absolutely conserved within each branch (Fig. S10*B*). Specifically, Protista exhibits Glu at position 46, Eumycetes and Plantae demonstrate Thr, while Animalia possesses Leu (Fig. S10*B*). This progressive alteration of residue 46 reveals an evolutionary pattern transitioning from a negatively charged amino acid to a polar amino acid and ultimately to a hydrophobic amino acid. These findings suggest that Ykt6 adopts a more compact conformation in advanced organisms, allowing for conformational regulation.

## Experimental procedures

### Protein preparation

Genes of *y*Ykt6ΔC (1–195) and *r*Ykt6ΔC (1–193) were individually cloned into a pET-M3C vector containing a His6 tag. All the mutations were generated using the standard PCR-based method and confirmed by DNA sequencing. Proteins were expressed in *Escherichia coli* BL21 (DE3) host cells at 16 °C overnight and purified by using a Ni^2+^–NTA agarose affinity chromatography followed by SEC in a normal buffer (50 mM Tris pH 8.0, 100 mM NaCl, and 1 mM EDTA).

### Fluorescent labeling of protein

The 25 μM double cysteine mutants of *y*Ykt6 or *r*Ykt6 proteins were incubated with 100 μM donor (Alexa Fluor 555 Maleimide, Thermo Fisher Scientific Inc), and 200 μM acceptor (Alexa Fluor 647 Maleimide, Thermo Fisher Scientific Inc) in the presence of 1 mM Tris (2-carboxyethyl) phosphine hydrochloride at 4 °C overnight. Afterward, the unreacted dyes were removed from the protein solution by SEC using a normal buffer containing 1 mM β-mercaptoethanol.

### smFRET experiments and data analysis

The coverslip and slide were cleaned and then coated with 0.1 mg/ml poly-lysine-PEG-NTA (PLL(20)-g[3.5]-PEG(2)-NTA, SuSoS AG Inc) to prevent nonspecific adsorption of proteins to the surface (26). The his-tagged protein was immobilized on the surface by Ni^2+^-NTA affinity. Single-molecule fluorescence images were taken by using a home-built wide field fluorescence imaging system (27). A 532 nm laser was used to excite the donor and generate FRET. A dual viewer (OptoSplit II, Andor Technology Plc) was used to separate the fluorescence of different colors emitted by donor and acceptor respectively, and then the fluorescence image was taken by an EMCCD camera (iXon 897, Andor Technology Plc) with an exposure time of 100 ms.

The temporal traces of the fluorescence intensity of individual molecules of the donor and acceptor were extracted from the recorded series of images by using the ISMS software (http://isms.au.dk) (MATLAB encoded) (28). We determined the minimum number of conformational states by performing the analysis of Bayesian inference of smFRET trace using vbFRET and comparing the calculated mean log evidence (21). Afterward, the HMM analysis was performed by using the vbFRET software to recognize the change point of transitions in smFRET trajectories and meanwhile generated the ideal trajectories (HMM FRET). To count the probabilities of different conformational states, we performed a threshold analysis on the ideal trajectories (29, 30, 31), where the thresholds for each state were read from the transition density plot (initial *versus* final HMM FRET efficiency of every transition) that compiled all transitions. Histograms of raw FRET were obtained by combining all time points of each raw FRET trajectories and histograms of different states were obtained by combining all time points of each state FRET trajectories obtained by threshold analysis.

### Isothermal titration calorimetry

ITC measurements were performed on a MicroCal PEAQ-ITC at 25 °C. All protein samples were dissolved in a buffer condition containing 50 mM Tris (pH 8.0), 100 mM NaCl, 1 mM EDTA, and 1 mM β-mercaptoethanol. The titrations were carried out by injecting 40 μl aliquots of DPC (0.5 mM) into 280 μl aliquots of Ykt6 proteins (0.05 mM) at time intervals of 2 min to ensure that the titration peak returned to the baseline. The titration data were analyzed using the Malvern MicroCal PEAQ-ITC analysis program.

### Cell culture, imaging, and Western blot

HeLa cells and 293T cells were cultured in Dulbecco's modified Eagle's medium supplemented with 10% fetal bovine serum. The N-terminal GFP-tagged WT or mutant Ykt6 cloned into the pEGFP-C1 vector were individually introduced into cells by the lipofectamine transfection method. After 36 h transfection, the cells were imaged with a Leica dmi8 inverted fluorescence microscope. After 36 h transfection, 293T cells expressed with the GFP-tagged WT Ykt6 or its mutants were washed with PBS buffer, and then lysed with radioimmunoprecipitation assay lysis buffer (50 mM Tris pH 7.4, 150 mM NaCl, 1% Triton X-100, 1% sodium deoxycholate, 0.1% SDS, and 1 mM PMSF) on ice for 30 min. The resulting cell mixture was then centrifuged at 13,000 rpm for 30 min to separate the cytosol and membrane fractions. Equal amount of protein loading was shown by the total GFP-tagged Ykt6 or its mutants detected by Western blot using the anti-GFP antibody.

### SMD simulation

SMD simulation was performed using the NAMD 2.14 software package (32). We chose the constant-velocity stretching method to simulate the conformational opening process. The constant-velocity mode of SMD requires specification of SMD atom, which is linked by a spring to a virtual atom that moves at a constant velocity. The external steering potential energy and the steer force of SMD are defined as follows:

(1) $$U=\frac{1}{2}k{\left[vt-\left(\vec{r}-\vec{r_{0}}\right)\cdot \vec{n}\right]}^{2}$$

(2) $$\vec{F}=-\nabla U$$

where *U* is the steering potential energy, *k* is the spring constant, *v* is the pulling rate, *t* is time, $\vec{r}$ is the instantaneous position of the SMD atom (or the centroid of the SMD atoms), $\vec{r_{0}}$ is the initial position of the SMD atom (or centroid of the SMD atoms), and $\vec{n}$ is the unit vector of the pulling direction.

SMD simulations were performed for three systems: WT *r*Ykt6ΔC, WT *y*Ykt6ΔC, and *y*Ykt6ΔC-T46LQ57A mutant. The initial structure of WT *r*Ykt6 was adapted from the crystal structure of *r*Ykt6ΔC/DPC (Protein Data Bank-ID: 3KYQ), and the initial conformation of WT *y*Ykt6 was obtained from the UniProt database (Name: P36015 Ykt6_YEAST, Structure method: AlphaFold) (23). The initial structure of *y*Ykt6-T46L/Q57A mutant system was mutated based on WT *y*Ykt6 by using the PyMOL molecular graphics program (http://www.pymol.org/).

A typical system was established with visual molecular dynamics (33) containing ∼90,000 atoms in the cubic simulation box measured ∼100 × 100 × 100 Å^3^. The protein and lipid were modeled using the CHARMM27 force field (34), while the water molecules were represented using the TIP3P model (35). The protein was solvated in a water box and subsequently neutralized by the addition of Na^+^ and Cl^−^ ions, resulting in a salt concentration of 100 mM. The 1 to 4 parameters specified that all 1 to 3 pairs will be excluded along with all pairs connected by a set of two bonds of bonded atoms from nonbonded interactions, and we set a nonbonded pair list within 13.5 Å. The electrostatic interactions for such pairs are modified by this constant factor. van der Waals interactions were smoothed beyond 10 Å and truncated at 12 Å. Nonbonded and electrostatic interactions were calculated at every time step. Berendsen pressure bath coupling method (36) is used to realize coupling to 1.01325 bar. The Langevin thermostat was employed to maintain the system at 298 K and the damping constant was set as 5/ps. The ionized system was energy-minimized by 20,000 steps, following a 1 ns equilibration run with constant temperature and a 2 ns equilibration run with constant pressure.

In the FixLsmdS mode for steered dynamics simulation implemented in the simulation package NAMD, we selected the backbone atoms and C_β_ atoms of longin domain (*r*Ykt6 residue 1–131, *y*Ykt6 residue 1–133) as fixed atoms, and the external force was applied to the backbone atoms and CB atoms of SNARE (*r*Ykt6 residue 132–198, *y*Ykt6 residue 134–200), which pulled SNARE along the vector from centroid of the longin domain toward the centroid of SNARE core (22).

The force constant was set to 5 (kcal/mol)/Å^2^ and the velocity of the movement of SMD reference position was set to 1.5 Å/ns. Each SMD simulation lasted for 20 ns in NVT ensemble. Coordinates were saved every 2 fs.

### Sequence analysis

To identify Ykt6 orthologs, a comprehensive search was initiated by employing BLASTP algorithm (National Center for Biotechnology Information; http://blast.ncbi.nlm.nih.gov) (37). The search focused on identifying Ykt6 orthologs from closely related organisms, utilizing prominent databases such as UniProtKB/Swiss-Prot (swissprot) and Model Organisms (landmark). To classify the prototypical Ykt6 family proteins, a sequence alignment was performed using the MEGA11 software (https://megasoftware.net), employing default parameters (38). The ensuing phylogenetic trees were computed in MEGA11 utilizing the Neighborhood Joining algorithm, and bootstrapping with 1000 iterations was applied when deemed necessary. The resulting phylogenetic trees were visually depicted using iToL: Interactive Tree of Life (https://itol.embl.de) for enhanced clarity and interpretation (39).

## Data availability

All experimental data are contained within the article and the Supporting Information. The full sequence alignment data will be made available upon request.

## Supporting information

This article contains supporting information.

## Conflict of interest

The authors declare that they have no conflicts of interest with the contents of this article

## References

[1] Weber T., Zemelman B.V., McNew J.A., Westermann B., Gmachl M., Parlati F. (1998). SNAREpins: minimal machinery for membrane fusion. Cell.

[2] Poirier M.A., Xiao W., Macosko J.C., Chan C., Shin Y.K., Bennett M.K. (1998). The synaptic SNARE complex is a parallel four-stranded helical bundle. Nat. Struct. Biol..

[3] Söllner T.H., Rothman J.E. (1996). Molecular machinery mediating vesicle budding, docking and fusion. Cell Struct. Funct..

[4] Sutton R.B., Fasshauer D., Jahn R., Brunger A.T. (1998). Crystal structure of a SNARE complex involved in synaptic exocytosis at 2.4 A resolution. Nature.

[5] Jahn R., Scheller R.H. (2006). SNAREs--engines for membrane fusion. Nat. Rev. Mol. Cell Biol..

[6] Rapaport D., Fichtman B., Weidberg H., Sprecher E., Horowitz M. (2018). NEK3-mediated SNAP29 phosphorylation modulates its membrane association and SNARE fusion dependent processes. Biochem. Biophys. Res. Commun..

[7] Rossi V., Banfield D.K., Vacca M., Dietrich L.E., Ungermann C., D'Esposito M. (2004). Longins and their longin domains: regulated SNAREs and multifunctional SNARE regulators. Trends Biochem. Sci..

[8] Daste F., Galli T., Tareste D. (2015). Structure and function of longin SNAREs. J. Cell Sci..

[9] Pylypenko O., Schönichen A., Ludwig D., Ungermann C., Goody R.S., Rak A. (2008). Farnesylation of the SNARE protein Ykt6 increases its stability and helical folding. J. Mol. Biol..

[10] Veit M. (2004). The human SNARE protein Ykt6 mediates its own palmitoylation at C-terminal cysteine residues. Biochem. J..

[11] Fukasawa M., Varlamov O., Eng W.S., Söllner T.H., Rothman J.E. (2004). Localization and activity of the SNARE Ykt6 determined by its regulatory domain and palmitoylation. Proc. Natl. Acad. Sci. U. S. A..

[12] Tochio H., Tsui M.M., Banfield D.K., Zhang M. (2001). An autoinhibitory mechanism for nonsyntaxin SNARE proteins revealed by the structure of Ykt6p. Science.

[13] Wen W., Yu J., Pan L., Wei Z., Weng J., Wang W. (2010). Lipid-induced conformational switch controls fusion activity of longin domain SNARE Ykt6. Mol. Cell.

[14] Wu S., Wang D., Weng J., Liu J., Wang W. (2018). A revisit of the conformational dynamics of SNARE protein rYkt6. Biochem. Biophys. Res. Commun..

[15] Karuna M.P., Witte L., Linnemannstoens K., Choezom D., Danieli-Mackay A., Honemann-Capito M. (2020). Phosphorylation of Ykt6 SNARE domain regulates its membrane recruitment and activity. Biomolecules.

[16] Barz S., Kriegenburg F., Henning A., Bhattacharya A., Mancilla H., Sánchez-Martín P. (2020). Atg1 kinase regulates autophagosome-vacuole fusion by controlling SNARE bundling. EMBO Rep..

[17] McGrath K., Agarwal S., Tonelli M., Dergai M., Gaeta A.L., Shum A.K. (2021). A conformational switch driven by phosphorylation regulates the activity of the evolutionarily conserved SNARE Ykt6. Proc. Natl. Acad. Sci. U. S. A..

[18] McNew J.A., Sogaard M., Lampen N.M., Machida S., Ye R.R., Lacomis L. (1997). Ykt6p, a prenylated SNARE essential for endoplasmic reticulum-Golgi transport. J. Biol. Chem..

[19] Hasegawa H., Yang Z., Oltedal L., Davanger S., Hay J.C. (2004). Intramolecular protein-protein and protein-lipid interactions control the conformation and subcellular targeting of neuronal Ykt6. J. Cell Sci..

[20] Hasegawa H., Zinsser S., Rhee Y., Vik-Mo E.O., Davanger S., Hay J.C. (2003). Mammalian ykt6 is a neuronal SNARE targeted to a specialized compartment by its profilin-like amino terminal domain. Mol. Biol. Cell.

[21] Bronson J.E., Fei J., Hofman J.M., Gonzalez R.L., Wiggins C.H. (2009). Learning rates and states from biophysical time series: a bayesian approach to model selection and single-molecule FRET data. Biophys. J..

[22] Weng J., Yang Y., Wang W. (2015). Lipid regulated conformational dynamics of the longin SNARE protein Ykt6 revealed by molecular dynamics simulations. J. Phys. Chem. A..

[23] Consortium U. (2023). UniProt: the universal protein knowledgebase in 2023. Nucleic Acids Res..

[24] Veltman D.M., Insall R.H. (2010). WASP family proteins: their evolution and its physiological implications. Mol. Biol. Cell.

[25] Dey S., Zhou H.X. (2023). Why does synergistic activation of WASP, but Not N-WASP, by Cdc42 and PIP(2) Require Cdc42 Prenylation?. J. Mol. Biol..

[26] Zhen G., Zurcher S., Falconnet D., Xu F., Kuennemann E., Textor M. (2005). NTA-Functionalized Poly(L-lysine)-g-Poly(Ethylene Glycol): a polymeric interface for binding and studying 6 his-tagged proteins. Conf. Proc. IEEE Eng. Med. Biol. Soc..

[27] Feng Y., Zhang L., Wu S., Liu Z., Gao X., Zhang X. (2016). Conformational Dynamics of apo-GlnBP revealed by experimental and computational analysis. Angew. Chem. Int. Ed Engl..

[28] Preus S., Noer S.L., Hildebrandt L.L., Gudnason D., Birkedal V. (2015). iSMS: single-molecule FRET microscopy software. Nat. Methods.

[29] McKinney S.A., Déclais A.C., Lilley D.M., Ha T. (2003). Structural dynamics of individual Holliday junctions. Nat. Struct. Biol..

[30] Fei J., Kosuri P., MacDougall D.D., Gonzalez R.L. (2008). Coupling of ribosomal L1 stalk and tRNA dynamics during translation elongation. Mol. Cell.

[31] McKinney S.A., Joo C., Ha T. (2006). Analysis of single-molecule FRET trajectories using hidden markov modeling. Biophys. J..

[32] Phillips J.C., Braun R., Wang W., Gumbart J., Tajkhorshid E., Villa E. (2005). Scalable molecular dynamics with NAMD. J. Comput. Chem..

[33] Humphrey W., Dalke A., Schulten K. (1996). VMD: visual molecular dynamics. J. Mol. Graph..

[34] Sapay N., Tieleman D.P. (2011). Combination of the CHARMM27 force field with united-atom lipid force fields. J. Comput. Chem..

[35] Jorgensen W.L., Chandrasekhar J., Madura J.D., Impey R.W., Klein M.L. (1983). Comparison of simple potential functions for simulating liquid water. J. Chem. Phys..

[36] Berendsen H.J.C., Postma J.P.M., van Gunsteren W.F., DiNola A., Haak J.R. (1984). Molecular dynamics with coupling to an external bath. J. Chem. Phys..

[37] Altschul S.F., Gish W., Miller W., Myers E.W., Lipman D.J. (1990). Basic local alignment search tool. J. Mol. Biol..

[38] Kumar S., Stecher G., Li M., Knyaz C., Tamura K. (2018). MEGA X: molecular evolutionary genetics analysis across computing platforms. Mol. Biol. Evol..

[39] Letunic I., Bork P. (2021). Interactive Tree Of Life (iTOL) v5: an online tool for phylogenetic tree display and annotation. Nucleic Acids Res..

