# Supplementary material for: Different conformational dynamics of SNARE protein Ykt6 among yeast and mammals
Source: J Biol Chem. 2023 Jun 26;299(8):104968. doi: 10.1016/j.jbc.2023.104968 (PMC10388204; doi:10.1016/j.jbc.2023.104968)
Supplement: Supporting Tables S1 and S2 and Figures S1–S10 [file mmc1.docx]

**Supporting Information**

**Different conformational dynamics of SNARE protein Ykt6 among yeast and mammals**

Jie Ji^1,#^, Yiping Yu^1,#^, Shaowen Wu^2,^, Dongdong Wang^3^, Jingwei Weng^1,*^, Wenning Wang^1,^*

**Table S1.** FRET efficiencies and proportions of various states of rat and yeast Ykt6

| States and FRET efficiency of *r*Ykt6 | Population | States and FRET efficiency of *y*Ykt6 | Population |
| --- | --- | --- | --- |
| E1– 0.2 | 31.4% | E1– 0.20 | 50.7% |
| E2– 0.35 | 19.8% | E2– 0.42 | 39.7% |
| E3– 0.48 | 19.3% | E3– 0.68 | 10.2% |
| E4– 0.64 | 16.2% |  |  |
| E5– 0.79 | 13.3% |  |  |

**Table S2**. Proportions of different states of WT *y*Ykt6 and its mutants

| states of *y*Ykt6 | Wild type | T46L/Q57A | T46L/Q57A-DPC | S176D |
| --- | --- | --- | --- | --- |
| E1 | 50.7% | 40.5% | 15.3% | 45.4% |
| E2 | 39.7% | 43.9% | 48.5% | 37.0% |
| E3 | 10.2% | 15.6% | 36.2% | 17.6% |

**
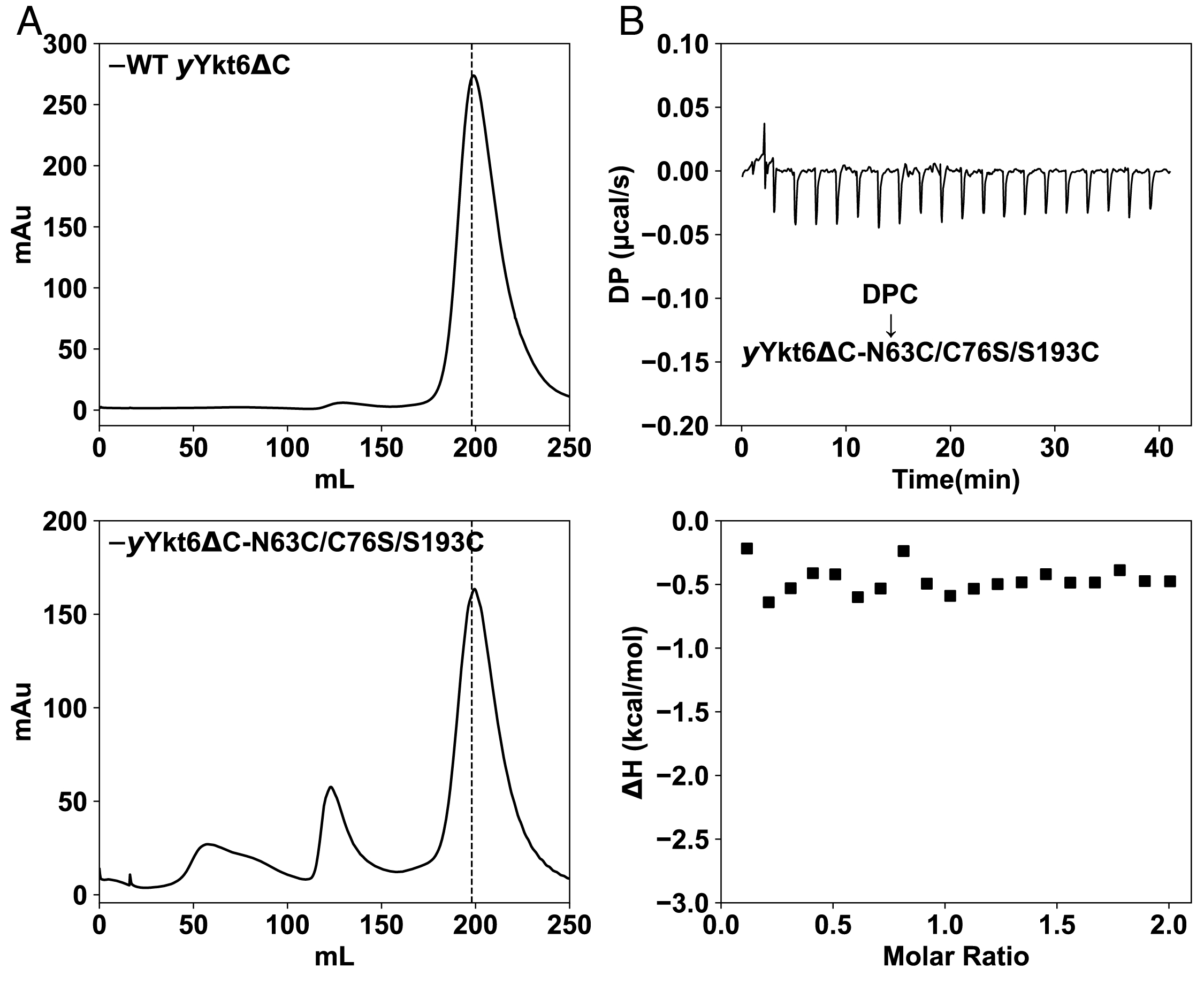
**

**Figure S1. Purified *y*Ykt6ΔC-N63C/C76S/S193C showed similar biochemical features to that of WT *y*Ykt6ΔC.** (A) Purified WT *y*Ykt6ΔC and *y*Ykt6ΔC-N63C/C76S/S193C proteins show similar elute volumes on SEC (AKTA FPLC system (GE Healthcare), Superdex 75 pg column). (B) ITC measurements of the interaction between *y*Ykt6ΔC-N63C/C76S/S193C and DPC.


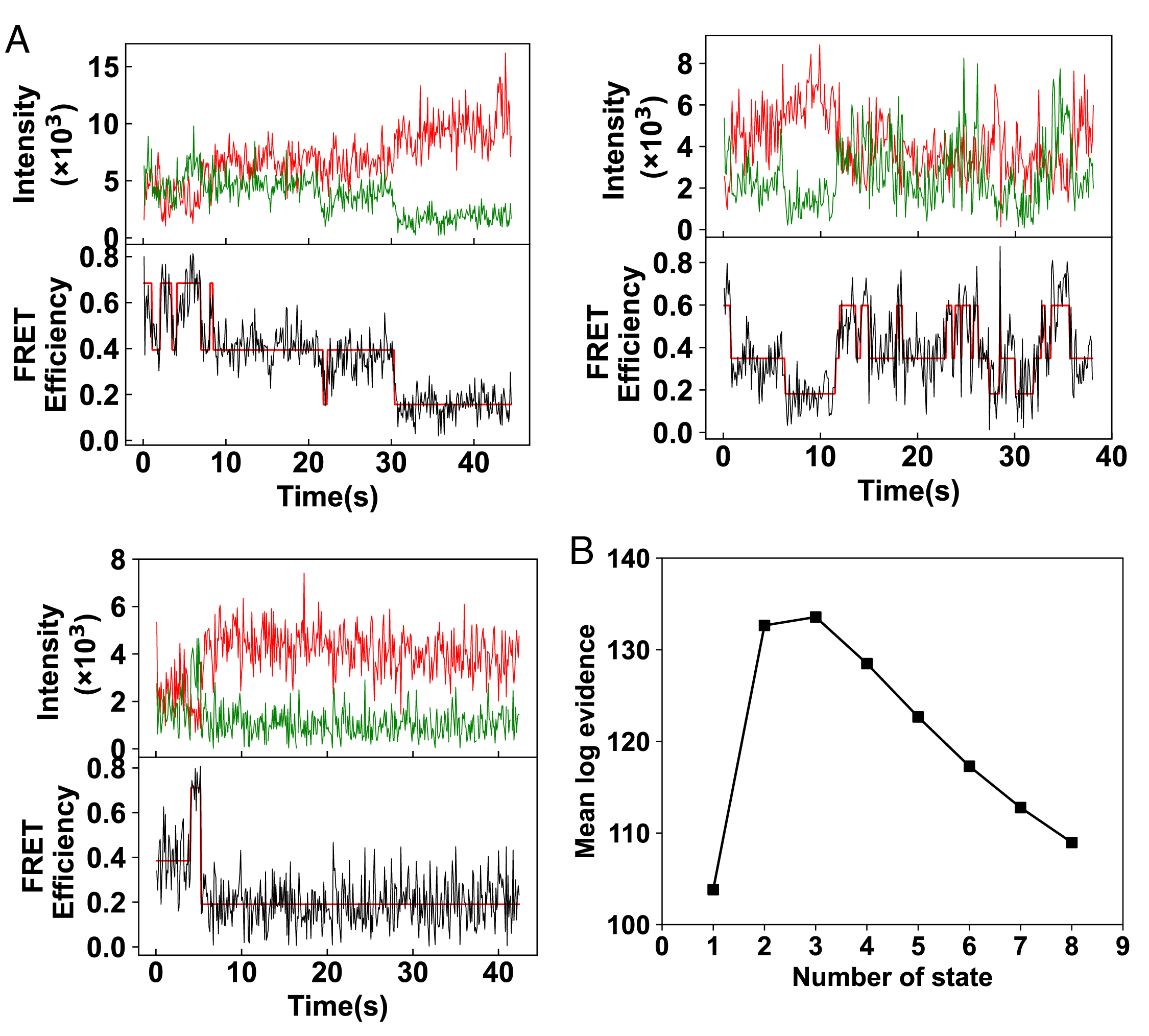


**Figure S2. Sample smFRET trajectories of *y*Ykt6ΔC.** (A) Three representative FRET trajectories and idealized HMM trajectory of *y*Ykt6ΔC showing multiple distinct efficiency levels. (B) Mean log evidence values of *y*Ykt6ΔC against number of states given by vbFRET. The minimum number of conformational states that given by vbFRET is three.


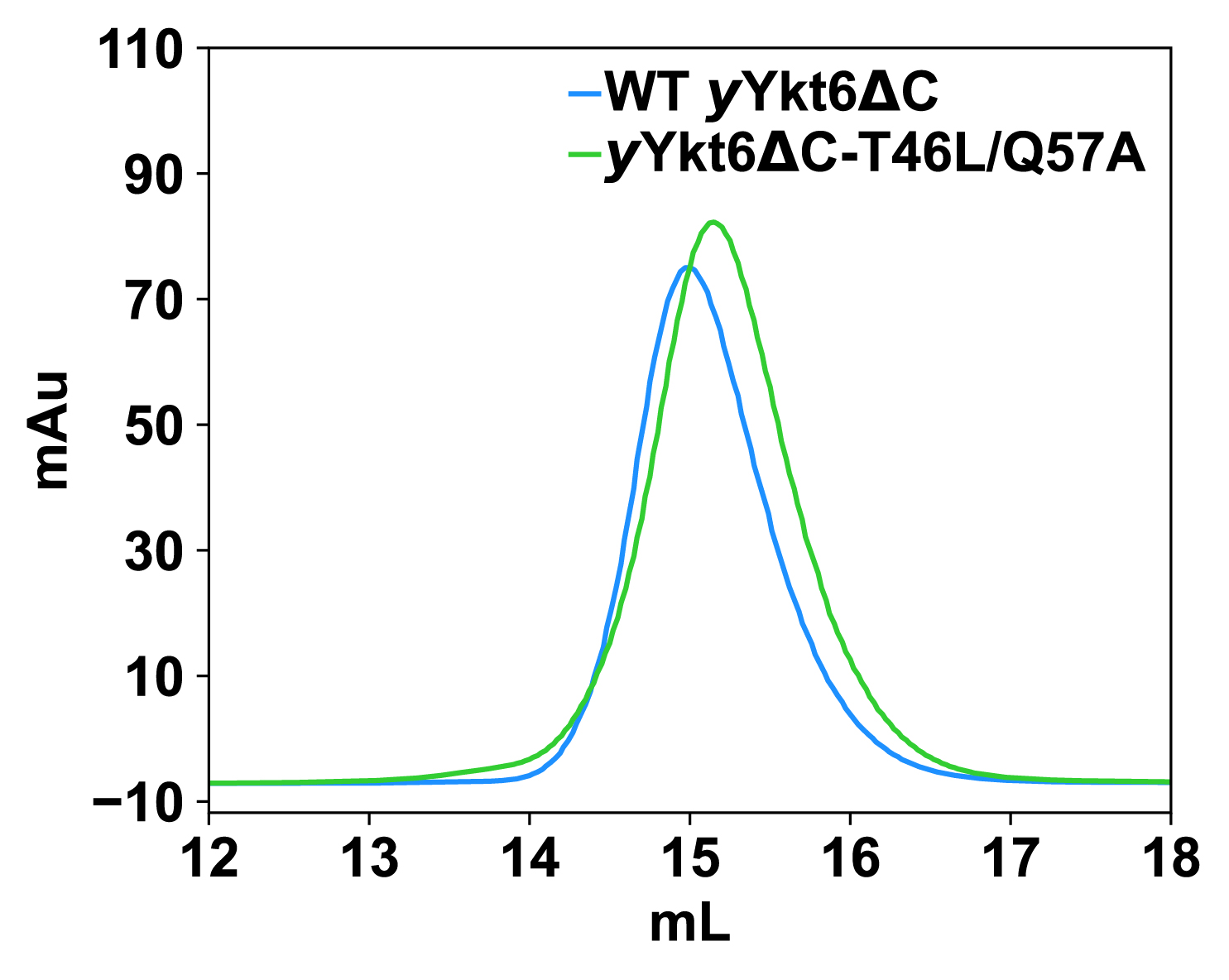


**Figure S3.** Analytical gel-filtration profiles of WT *y*Ykt6ΔC (blue) and *y*Ykt6ΔC T46L/Q57A (green).


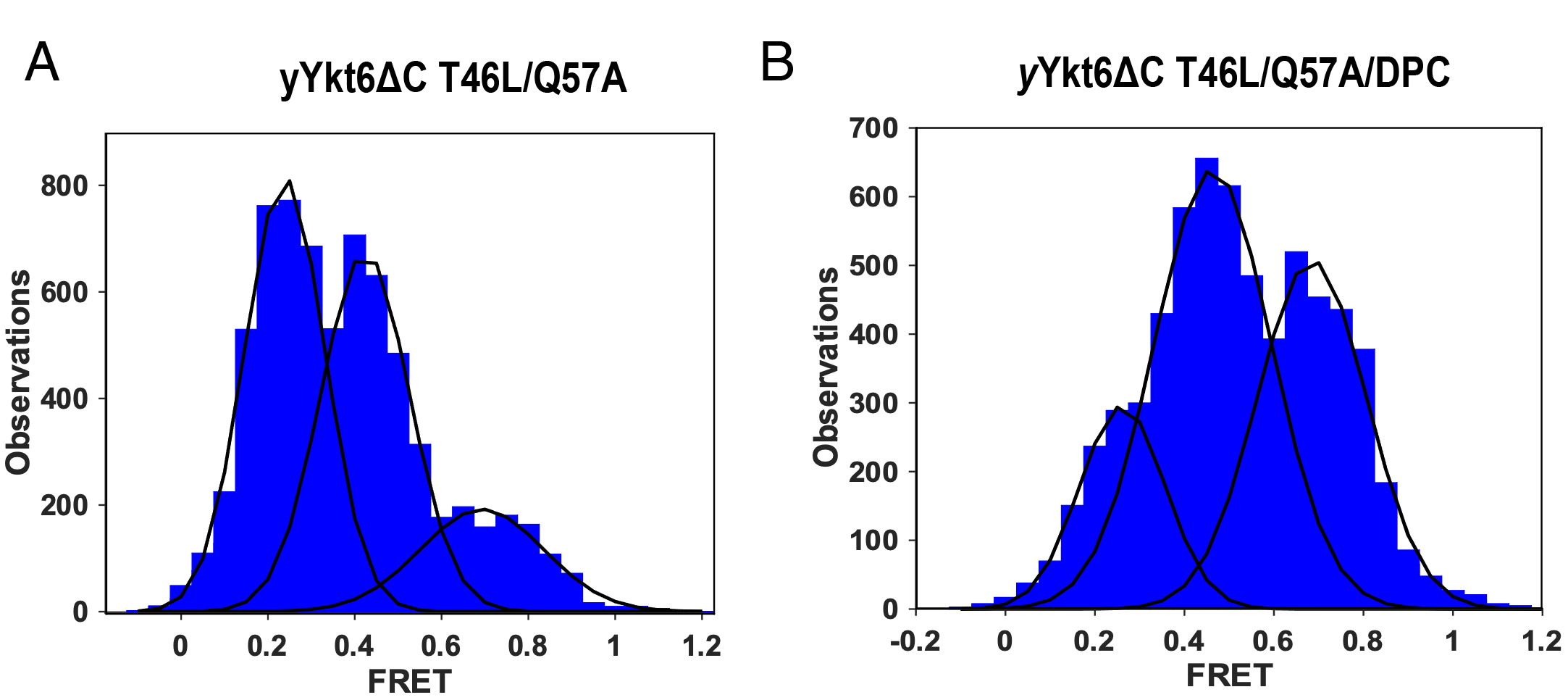


**Figure S4.** FRET efficiency histogram of *y*Ykt6ΔC T46L/Q57A (A) and *y*Ykt6ΔC T46L/Q57A/DPC (B) based on HMM analysis.


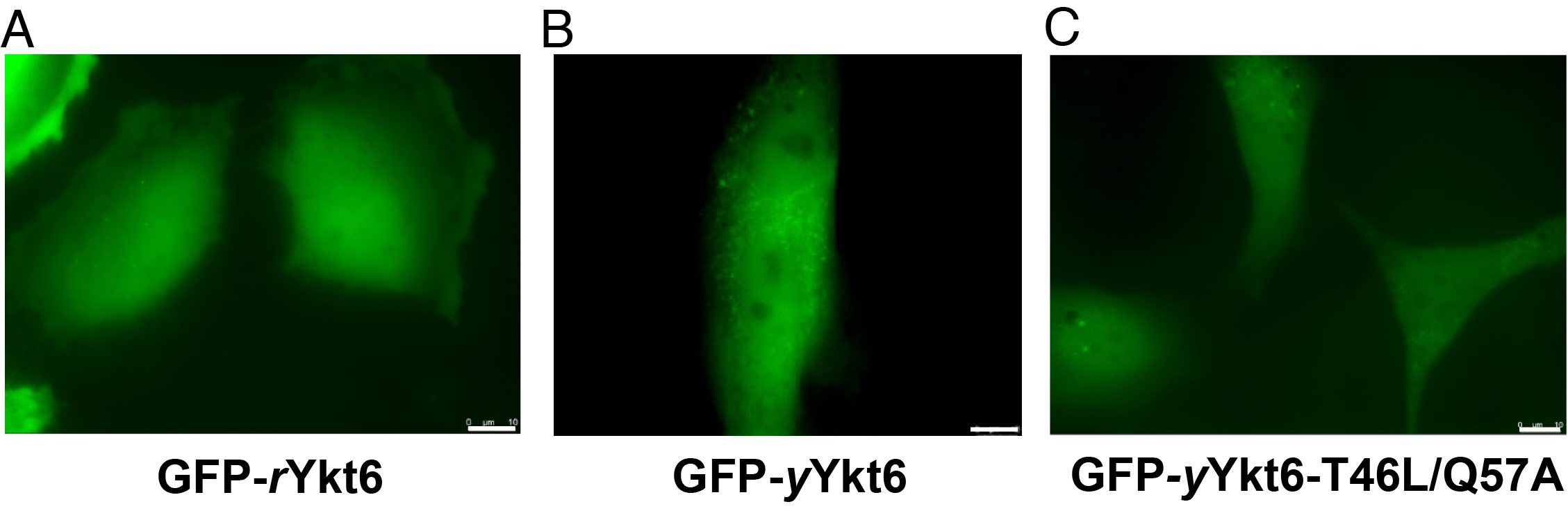


**Figure S5.** Intracellular localizations of GFP-*r*Ykt6 (A), GFP-*y*Ykt6 (B) and GFP-*y*Ykt6 T46L/Q57A (C) (Scale bar, 10 μm.)


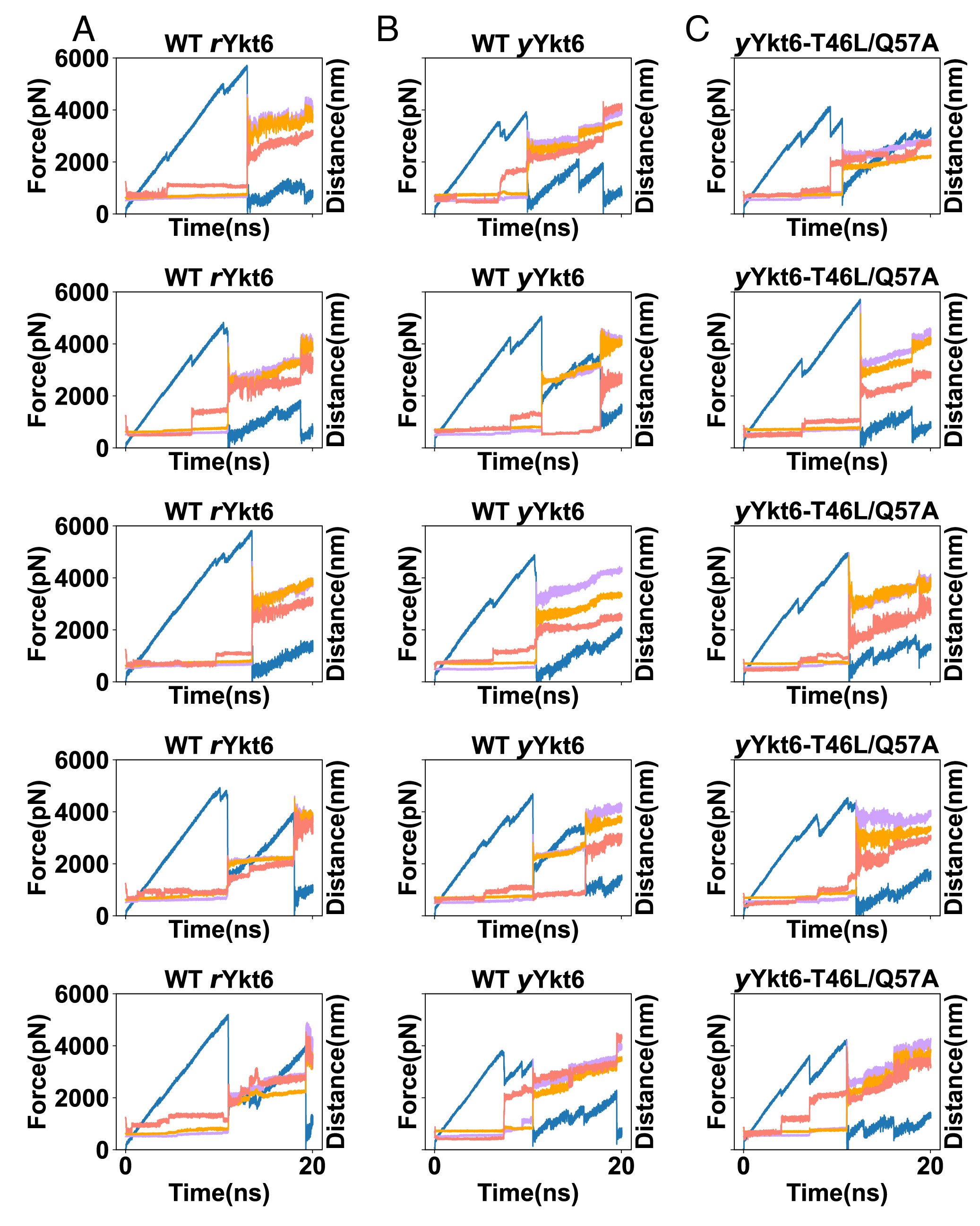


**Figure S6.** Time variations of SMD force (steel blue), inter-residue distances *d*_46-170_ (purple), *d*_46-167_ (orange), *d*_63-191_ (pink) based on five parallel trajectories of WT *r*Ykt6 （A）, WT *y*Ykt6 （B）, *y*Ykt6-T46L/Q57A (C) systems, respectively.


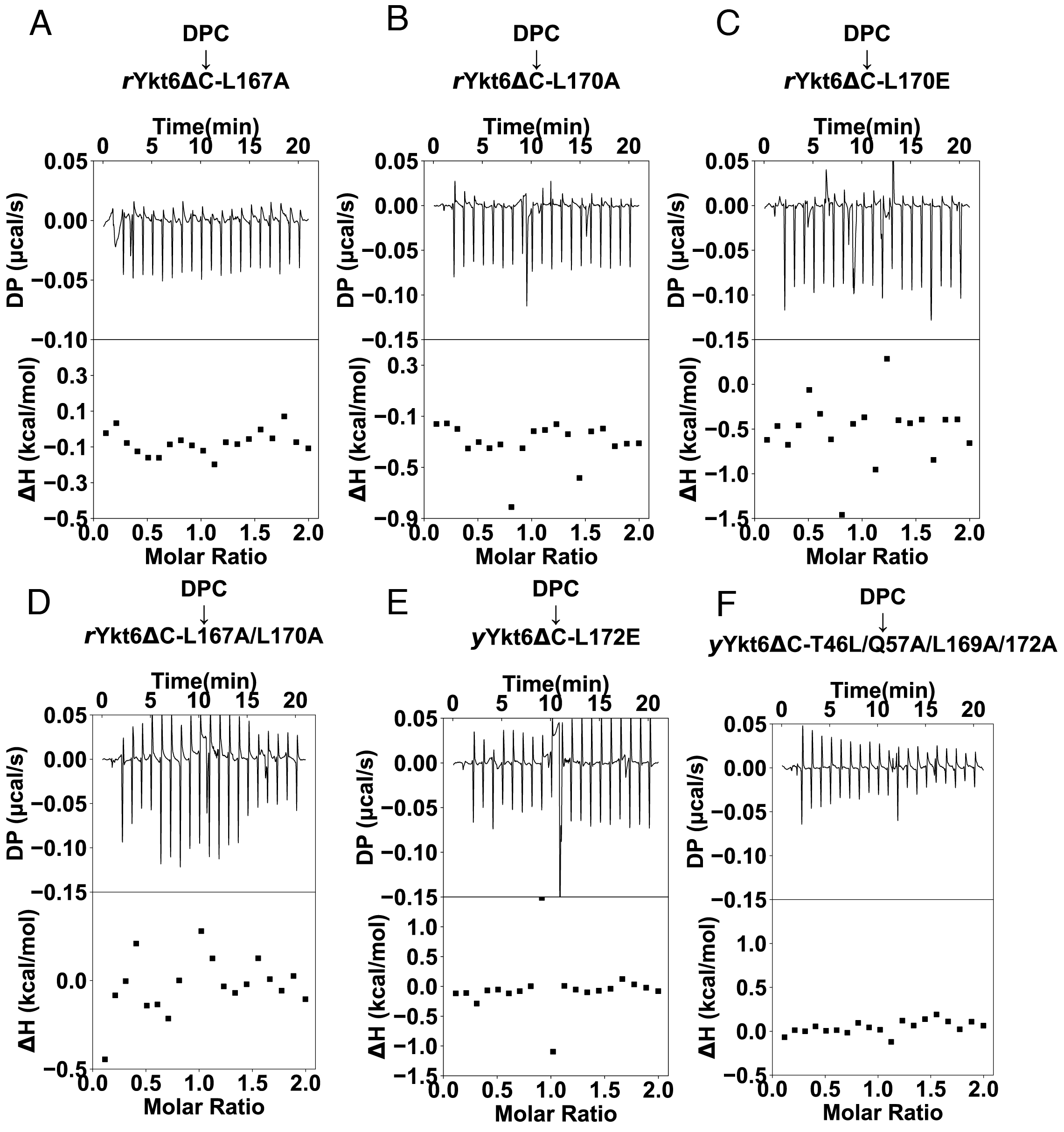


**Figure S7.** ITC measurements of the interaction between Ykt6ΔC-mutants and DPC.


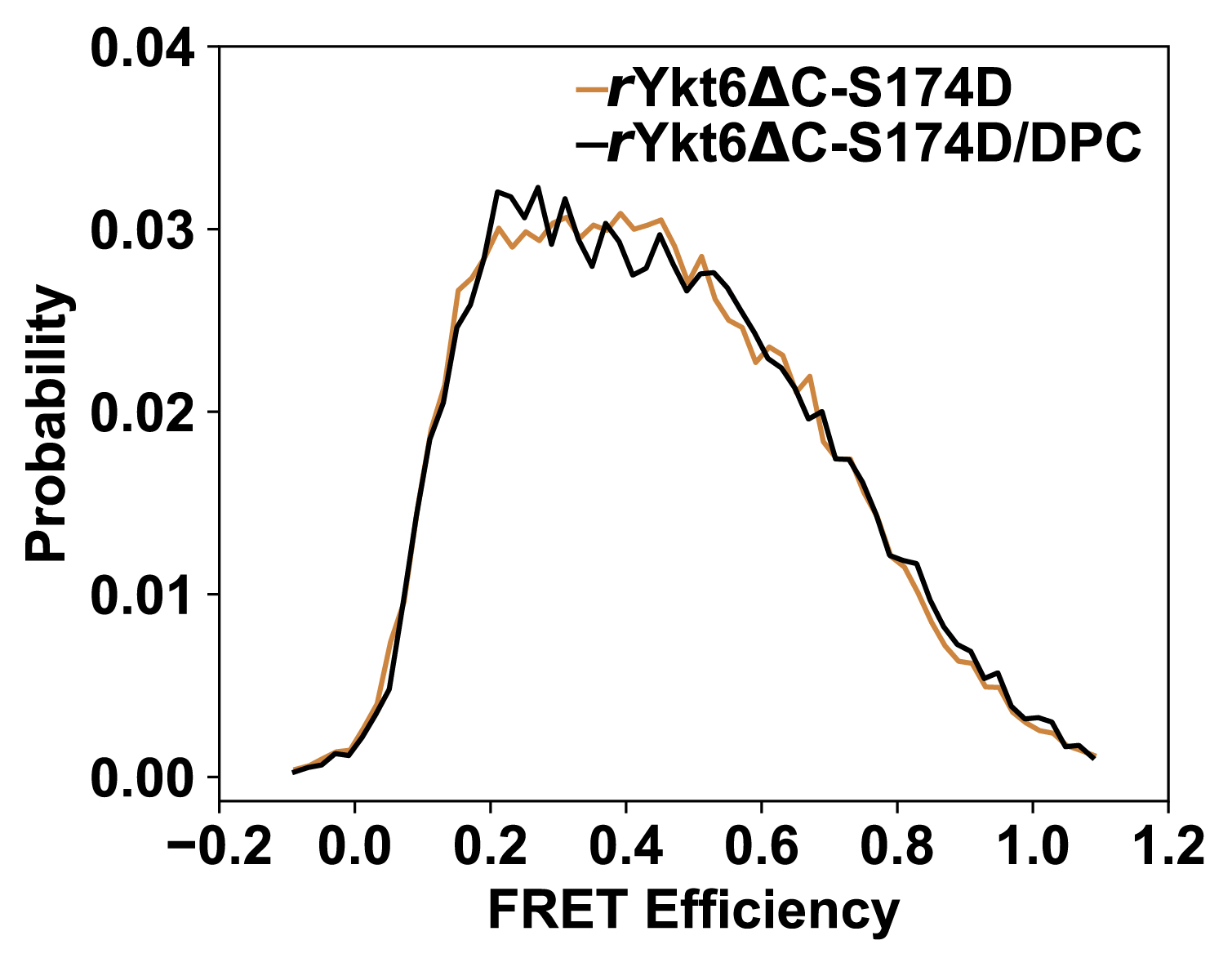


**Figure S8.** FRET efficiency distribution profiles of *r*Ykt6ΔC-S174D (peru) and *r*Ykt6ΔC-S174D/DPC (black).


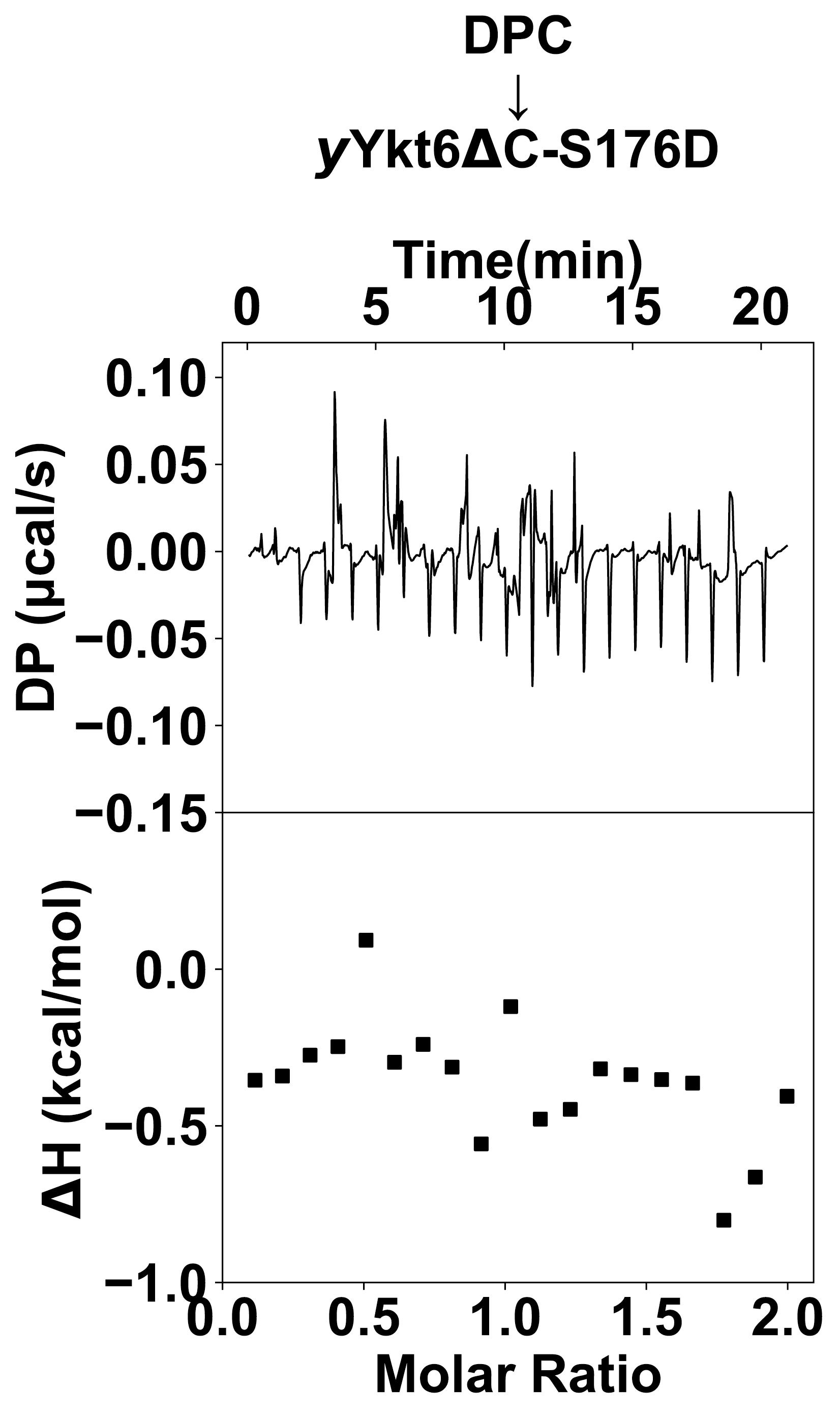


**Figure S9.** ITC measurements of the interaction between *y*Ykt6ΔC-S176D and DPC.


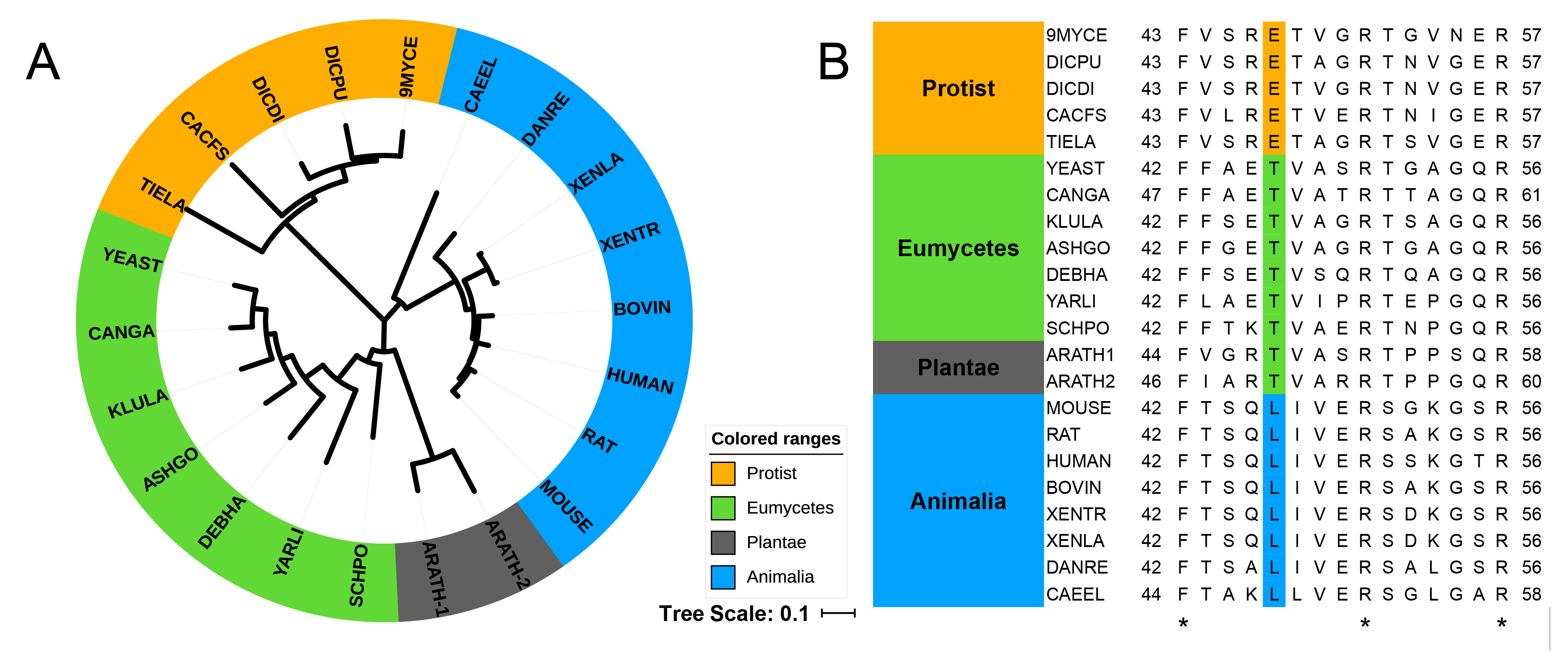


**Figure S10**. The evolutionary trend of eukaryotic Ykt6 proteins. (A) The Ykt6 protein sequences from various species were aligned using MEGA11, and a distance tree was generated based on the alignment results. Species originating from different kingdoms were distinguished by using different colors to mark them. (B) Sequence alignment of Ykt6 orthologues. Identical amino acids are denoted with an asterisk (*). The vertical row of colored amino acids represents the position of T46 in yeast. The following species are represented by their respective abbreviations: 9MYCE (P. violaceum), DICPU (D. purpureum), DICDI (D. discoideum), CACFS (C. fasciculata), TIELA (T. lacteum), YEAST (S. cerevisiae), CANGA (C. glabrata), KLULA (K. lactis), ASHGO (A. gossypii), DEBHA (D. hansenii), YARLI (Y. lipolytica), SCHPO (S. pombe), ARATH-1/2 (A. thaliana), MOUSE (M. musculus), RAT (R. norvegicus), HUMAN (H. sapiens), BROVIN (B. taurus), XENTR (X. tropicalis), XENLA (X. laevis), DANRE (D. rerio), and CAEEL (C. elegans).
